# Synergistic Molecular Strategies for Targeting the Unfolded Protein Response in Cancer Therapy

**DOI:** 10.3390/ph19060941

**Published:** 2026-06-15

**Authors:** Ovanes Muradyan, Moudood Tahir, Victoria Sarafian

**Affiliations:** 1 Department of Medical Biology, Medical Faculty, Medical University of Plovdiv, 4002 Plovdiv, Bulgaria; 22111139@mu-plovdiv.bg; 2 Division of Molecular and Regenerative Medicine, Research Institute at Medical University of Plovdiv, 4002 Plovdiv, Bulgaria

**Keywords:** synergistic therapies, UPR, ER stress, cancer cells, proteasome inhibitors

## Abstract

Targeted synergistic therapies represent a rapidly developing branch of oncology. The emergence of novel targeting agents allows for modulation of an ever-larger set of cellular pathways. The Unfolded Protein Response (UPR) is a key element of cellular proteostasis that is significantly hyperactivated in a wide range of cancer cell types, especially those with high secretory activity. As cancer cells are especially vulnerable to endoplasmic reticulum stress (ER Stress), they become heavily dependent on UPR function to maintain homeostasis. A wide number of pharmacologic agents can stimulate the UPR and shift it from its initial pro-survival phase to the terminal pro-apoptotic phase. Key strategies include the use of UPR feedback inhibitors (e.g., GRP78 antagonists), direct pathway inhibitors targeting the PERK or IRE1α branches, and signal pathway modulators (e.g., TKIs and BTK inhibitors) that indirectly exacerbate proteotoxic stress. In this study we provide a mechanistic framework where we classify synergistic therapies based on their mechanism of action and explore how they influence ER Stress and UPR activation. Evidence synthesized from these studies suggests that synergistic combinations can overcome therapeutic resistance and selectively induce apoptosis in cancer cells characterized by high proteotoxic stress.

## 1. Introduction

Many cancers exhibit increased levels of protein synthesis. Plasma membrane proteins and proteins purposed for secretion are processed inside the endoplasmic reticulum (ER) [1,2]. ER-associated degradation (ERAD) is one of the main quality control mechanisms of cellular proteostasis. In physiological conditions, ERAD is responsible for the clearance of misfolded proteins [3]. When the capacity of the folding machinery fails to catch up with increased rates of translation, typical for cancer cells, endoplasmic reticulum stress (ER Stress) is generated. This leads to the activation of an ER-localized gene transcription pathway termed Unfolded Protein Response (UPR). Agents that target the UPR might be leveraged to induce apoptosis in various cancers.

### 1.1. The UPR Pathway

**Figure 1:**
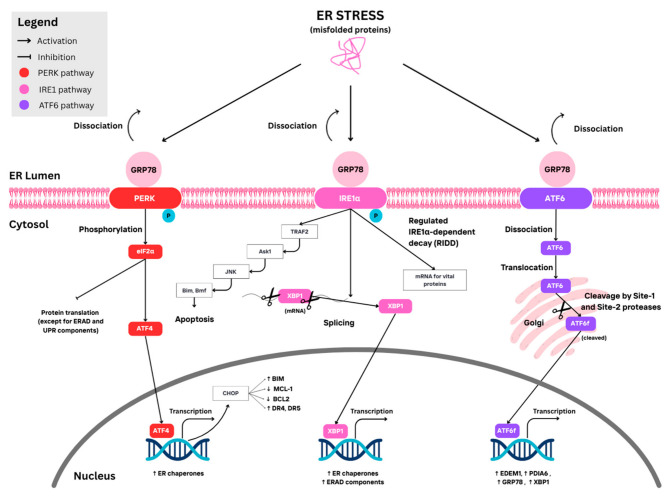
The UPR pathway. The figure illustrates the three main UPR receptors and the signaling branches they activate. The bottom shows the activation of the transcriptional programs that are specific to each branch.

The UPR consists of three branches as shown in Figure 1. They eventually trigger a complex transcriptional program, purposed to manage the ER Stress [4]. The UPR operates at two functional levels. UPR stage 1 exhibits a pro-survival function by inducing the transcription of chaperone and proteasome genes, which serve to alleviate the accumulation of misfolded proteins by either refolding or degrading them, respectively. If the cell fails to suppress the buildup of potentially toxic polypeptides and the ER Stress turns chronic, the nature of the UPR changes. In prolonged exposure to stress, pro-apoptotic gene products of the UPR start to increase significantly in concentration. This is accompanied by the degradation of key UPR mRNAs. UPR has now shifted into the pro-apoptotic stage 2, which ultimately drives the cell into apoptosis.

All three pathways are activated by sensors located in the ER membrane—PERK, IRE1α and ATF6. In their dormant state, they are associated with the GRP78 chaperone, also termed BiP (Binding immunoglobulin protein). GRP78 is coded by the Heat Shock Protein Family A (Hsp70) Member 5 gene (HSPA5). When misfolded proteins accumulate inside the ER, GRP78 dissociates from the sensors and contributes to the folding process. Simultaneously, the three membrane sensors are activated.

(A) Upon GRP78 dissociation, PERK oligomerizes and autophosphorylates. Then it proceeds to phosphorylate eukaryotic translation initiator factor 2α (eIF2α), thus inhibiting mRNA translation and protein synthesis. Exceptions to this are ERAD components, ER chaperones, UPR transcription factors, etc. [5]. Translation of mRNA for Activating Transcription Factor-4 (ATF4) is also selectively induced. In UPR stage 1, ATF4 stimulates the expression of ER chaperones and other proteins involved in the recovery of proteostasis. Additionally, ATF4 stimulates the expression of the pro-apoptotic C/EBP homologous protein (CHOP). In acute ER Stress, the elevation in CHOP levels is transient and has no significant effect. However, in chronic ER Stress, its concentration increases dramatically and facilitates the shift to UPR stage 2. Prolonged high levels of CHOP seem to have a key role in the execution of apoptosis [4,6].
(B) In similar fashion to PERK, when GRP78 dissociates, IRE1α undergoes oligomerization and autophosphorylation. IRE1α then performs splicing of the mRNA coding the X-box binding protein 1 (XBP1). The activated form—spliced XBP1, is a transcription factor. It enters the nucleus and proceeds to upregulate the transcription of genes, involved in ER protein folding and ERAD [7]. The transition from stage 1 to stage 2 UPR is marked by the activation of Regulated IRE1α-dependent decay (RIDD). RIDD is responsible for the degradation of the GRP78 mRNA and the mRNA of many other proteins involved in the negative regulation of apoptosis and cell growth [4]. In this way prolonged ER Stress proves itself as the pivotal point for the switch from the pro-survival to the pro-apoptotic UPR.
(C) ATF6 is the third membrane-bound ER Stress sensor. It exists as a dimer associated with GRP78. Upon GRP78 dissociation, ATF6 translocates from the ER to the Golgi apparatus where it is cleaved by Site-1 protease and Site-2 protease. Occasionally, the now active transcription factor ATF6f enters the nucleus and induces the expression of proteins, which either alleviate the ER Stress directly—degradation-enhancing α-mannosidase-like protein 1 (EDEM1), protein disulphide isomerase-associated 6 (PDIA6), or do so by influencing the UPR—expression of GRP78 and XBP1 [8].

### 1.2. The UPR Can Trigger Apoptosis

Apoptosis during stage 2 UPR is initiated by many simultaneous pathways that together push the cell towards self-destruction. For instance, high levels of CHOP suppress the expression of BCL2, BCL-XL, and MCL-1, while upregulating the expression of the pro-apoptotic BH3-only domain protein BIM [9]. CHOP also enhances the expression of Tribbles 3 (TRIB3)—a pseudokinase, responsible for the negative modulation of the Akt signaling pathway. Downstream AKT effectors downregulate the expression of caspase 3 and caspase 9. Moreover, AKT phosphorylates the pro-apoptotic BAD, thus inactivating it [10]. Hence, TRIB3 upregulation serves as a pro-apoptotic function. Finally, the PERK-ATF4-CHOP pathway upregulates the expression of the death receptor 4 (DR4) and death receptor 5 (DR5) [11].

The IRE1α pathway also has a significant contribution to apoptosis. Firstly, the IRE1α endonuclease activity is responsible for the so-called regulated IRE1α-dependent decay (RIDD), which leads to the cleavage of many mRNAs, essential for the normal cell functioning. These include GRP78 and many other substrates, crucial for the cell survival and proliferation [4,12]. Additionally, IRE1α has a kinase domain that associates with TNF receptor-associated factor 2 (TRAF2) and apoptosis signaling kinase 1 (ASK1). The newly formed complex phosphorylates c-Jun N-terminal kinase (JNK). JNK is responsible for the phosphorylation of the BH3-only Bim and Bmf which exhibit a pro-apoptotic function [13]. It also promotes the localization of Bad and Bax [14].

Although ATF6 serves primarily a pro-survival function, it could also indirectly contribute to apoptosis [15].

### 1.3. The UPR as a Therapeutic Target in Cancer

Most normal cells, both in vivo and in vitro do not experience ER Stress and therefore have very low levels of GRP78 and negligible levels of CHOP. When cultured in vitro healthy cells require artificial induction of ER Stress to experience higher GRP78 and CHOP. In contrast, most cancer cell types have chronically high levels of ER stress. A hallmark of this state is the increased level of GRP78, indicative of UPR activation. This pattern is particularly relevant to multiple myeloma (MM) and mantle-cell lymphoma (MCL), which are characterized by abnormal production of free light chains (FLCs) and Cyclin D1, respectively. This characteristic buildup of proteins and the subsequent ER Stress is the reason why proteasome inhibitors (PIs) are so effective in the treatment of these malignancies. Many other cancer types such as triple negative breast cancer [16] and prostate cancer are also notorious for their overexpression of proteins and proteotoxic stress. Cancer cells require the stage 1 UPR to maintain the concentration of misfolded proteins in the ER within limits compatible with cellular viability. This reliance on the UPR to maintain homeostasis could be exploited for the development of specific therapeutic strategies targeting cancer cells selectively, sparing normal cells.

The existing therapies are designed to amplify ER Stress and drive the UPR into stage 2 and thereby cause apoptosis. These include PIs, protein disulfide isomerase (PDI) inhibitors, ERAD inhibitors [17] and autophagy inhibitors. In addition, some other agents can target specific branches of the UPR pathway. Tyrosine kinase inhibitors (TKIs), conventional chemotherapeutics, ion transport and vesicle transport inhibitors are also proven to increase ER Stress and therefore cause activation of the UPR. In this line of thought combination treatments might be of particularly high efficiency. Synergism proves to be a powerful tool for driving the UPR from the pro-survival stage 1 to the pro-apoptotic stage 2. Additionally, it can overcome resistance in cells that are otherwise resistant to one of the used drugs when applied alone.

Despite the major increase in the success of the listed therapies, PIs, as well as many other agents, face obstacles such as resistance to therapy or insufficient induction of the UPR to drive the cancer cells into apoptosis. Synergistic therapies are often capable of overcoming resistance and stimulating the shift from the adaptive UPR to terminal UPR. In the current literature there is no unified framework that maps out the potential synergistic approaches targeting the UPR pathway. Establishing such a comprehensive framework serves as a critical roadmap that may contribute to the development of next-generation oncology protocols. By systematically mapping these interactions, potent combinations could be identified that effectively amplify ER Stress and UPR activation and successfully drive the cells into apoptosis or cytotoxic cell death.

The aim of this review is to categorize and evaluate the synergistic therapeutic strategies that leverage ER Stress-inducing agents to hyperactivate the UPR and trigger programmed cell death or cellular cytotoxicity. By classifying these synergistic approaches, we attempt to provide a more structured and clear insight into the mechanism via which cancers, characterized by proteotoxic stress, could potentially be targeted. To the best of our knowledge, this manuscript represents the first review attempting to address the topic of UPR-targeting therapies within the framework of synergistic mechanisms. Finally, we wanted to provide a critical analysis of the potential of the different therapeutic strategies.

## 2. Therapeutic Approaches

All reported synergistic approaches use therapeutic agents that can be functionally classified into two broad categories: Primary ER Stress Inducers and Accompanying Drugs. These can then be divided into subclasses based on the mechanism of action of the specific drug. While the ER Stress inducers’ action ultimately culminates into a common final effect—a direct disruption of proteostasis and an increase in the concentrations of misfolded proteins—the nature of action of the accompanying drug shows greater variety and nuances among the investigated therapies. Therefore, for the purposes of clear systematization, we present a brief overview of the used ER Stress inducers, followed by a categorization of the investigated therapeutic approaches based on the mode of action of the accompanying drug. The accompanying drugs were put into separate categories based on the following logic. If they target GRP78—the main feedback regulator—they were classified as UPR Feedback Inhibitors. All agents targeting different members of the UPR pathway except for GRP78 were classified as UPR Pathway Inhibitors. All drugs that inhibit or activate a key signaling pathway in the cell as their direct and explicit effect were put into the Signal Pathway Inhibitors and Signal Pathway Activators category, respectively. If the accompanying drug targeted a particular element of cellular proteostasis, it was categorized as a Proteostasis Inhibitor. Drugs that target ion transport were classified as Ion Transport Inhibitors. The category of classic chemotherapeutics includes agents that are considered conventional chemotherapeutics. Lastly, those accompanying drugs which did not fall into any of the defined categories were grouped as Others.

## 3. Primary ER Stress Inducers

**Figure 2:**
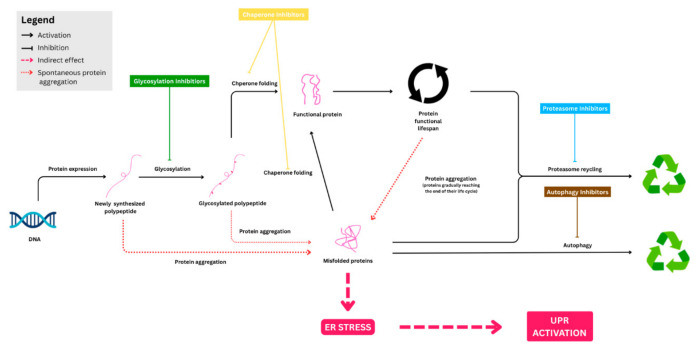
Classification of primary ER Stress inducers. The figure demonstrates the lifecycle of a polypeptide chain. To the left are illustrated the steps of protein synthesis and maturation and to the right is presented the process of protein recycling. To the bottom is depicted the logic of ER Stress accumulation and UPR activation. The four main categories of primary ER Stress inducers are presented, showing which part of the protein lifecycle they interfere in.

The currently reviewed primary ER Stress inducers may be categorized into a few separate and functionally distinct drug categories, based on their mechanism of action. This classification is visually summarized in Figure 2.

PIs represent a major class of ER Stress inducers, exerting their effects by reversibly binding to the catalytic β subunits—specifically β1, β2 and β5—of the 26S proteasome. This inhibition leads to the accumulation of proteotoxic aggregates within the cell. Canonically, this overwhelms the ER and leads to the activation of the UPR and subsequent apoptosis. PIs are a cornerstone in the treatment of hematologic malignancies such as multiple myeloma (MM) and mantle cell lymphoma (MCL) [18]. Despite their impressive effectiveness in the clinic, resistance has been a major obstacle, especially in MM. There are three FDA-approved PIs—bortezomib, ixazomib and carfilzomib. Additionally, oprozomib and MG132 are also used. Xanthohumol is another compound with attributed proteasome inhibition properties [19].

Glycosylation inhibitors serve to inhibit the N-linked glycosylation in the ER. This in turn leads to the rapid accumulation of misfolded proteins and ER Stress. The most used member of this class is 2-DG, which has several cellular targets—it is a competitive inhibitor of the enzyme phosphoglucose isomerase. Additionally, glycosyltransferases mistakenly use 2-DG instead of mannose for protein glycosylation. This results in the formation of defective sugar chains on proteins and occasionally prevents their adequate folding. DON is a potent inhibitor of GFAT (glutamine:fructose-6-phosphate amidotransferase). GFAT is a key member of the hexosamine pathway, and its inhibition ultimately leads to ER Stress. Another drug with an effect on glycosylation is the antibiotic tunicamycin. It is an inhibitor of UDP-N-acetylglucosamine–dolichol phosphate N-acetylglucosamine-1-phosphate transferase (DPAGT1), the enzyme that catalyzes the first step of N-linked glycosylation. Inhibition of this enzyme prevents the formation of lipid-linked oligosaccharide precursors, thereby blocking N-linked glycosylation of nascent proteins in the endoplasmic reticulum. This leads to accumulation of misfolded proteins, activation of the Unfolded Protein Response (UPR), and severe ER Stress. Tunicamycin, however, is extremely toxic to both healthy and cancer cells and is only used in lab settings.

The folding inhibitors category includes the compounds Onalespib, an HSP90 inhibitor and Quercetin, an HSP70 inhibitor. By inhibiting these chaperones, they directly reduce the potential of the protein folding machinery.

The autophagy inhibitors are another category with the main representatives in this review being 3-methyladenine and Antrocinol.

## 4. Classification of Synergistic Strategies by Mechanism of Action

### 4.1. Therapies Using UPR Feedback Inhibitors

**Figure 3:**
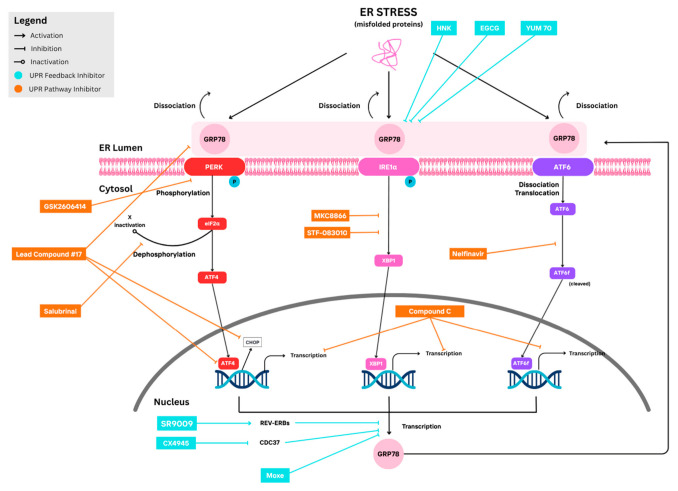
UPR feedback inhibitors and UPR pathway inhibitors. The diagram shows two distinct strategies for overcoming the cancer cell’s ability to survive chronic ER Stress. UPR feedback inhibitors (such as GRP78 inhibitors EGCG or YUM70 prevent the feedback suppression of the stress response, thereby causing UPR sensors to switch into an active state. UPR pathway inhibitors (such as PERK or IRE1α inhibitors) inhibit the pro-survival “coping mechanisms,” rendering it incapable of managing misfolded protein accumulation. Together, these approaches demonstrate how manipulating the UPR’s own regulatory architecture can shift the UPR from a pro-survival stage 1 to a pro-apoptotic stage 2.

The strategies mentioned in this category rely on enhancing the ER Stress while simultaneously inhibiting the action of GRP78, thus enhancing further UPR activation and driving the cells into apoptosis. Among the listed papers are several approaches that used a synergy between an ER Stress inducer and a UPR feedback inhibitor. They are depicted in blue in Figure 3 and are structured formally in Table 1.

A therapy that combines 2-DG, a glycolysis and glycosylation inhibitor, and Moxetumomab pasudotox (Moxe), a CD22-targeted immunotoxin that prevents the upregulation of GRP78, has been reported [20]. Blocking the upregulation of GRP78 effectively hinders the feedback inhibition of the pathway, rendering all the three UPR sensors perpetually active. Three of the investigated approaches rely on the green tea-derived compound EGCG (epigallocatechin-3-gallate), a compound that binds directly to the ATP-ase domain of GRP78 and inhibits its chaperone function, thus enhancing UPR activation. It can also be presumed that EGCG prevents GRP78 from effectively sequestering the UPR sensors, potentially further enhancing UPR activation. One of the aforementioned therapies explores the action of another plant compound HNK (honokiol) [21]. According to the findings in this paper, HNK has greater affinity for the nucleotide-binding domains of GRP78 than EGCG and caused double the levels of cell death in the neuroectodermal tumor cell lines compared to EGCG. Among the other two therapies using EGCG, one synergized with Quercetin and another with paclitaxel (Taxol) or vinblastine, respectively, for treatment of breast cancer cell lines [22,23].

Alternatively, downregulation of GRP78 may be achieved by altering the circadian clock of the tumor cells. In normal cells, this circadian rhythm regulates the 24 h transcription of genes with the control being established by two main proteins—CLOCK and BMAL1. Other major regulators are the PER and CRY proteins that antagonize CLOCK and BMAL1 and thus play key role in establishing the circadian clock’s rhythmicity. In contrast to normal cells, this machinery is disrupted in cancer cells. The nuclear receptors REV-ERBα and REV-ERBβ are two other members of this regulatory mechanism that serve to inhibit BMAL1. Wang et al. discovered that drug-induced activation of these receptors via the use of SR9009, a REV-ERBs agonist, resulted in downregulation of the key UPR regulator GRP78 and reduction in autophagy [24]. The team treated the MM cell lines RPMI-8226 and U266 with a combination of bortezomib and SR9009. They report synergistic enhancement of the tumor cell apoptosis.

Another approach in this category involves the use of CX-4945, a CK2-inhibitor, in synergy with bortezomib. CK-2 is a protein kinase responsible for the phosphorylation of multiple targets within the cell, among which is the co-chaperone Cdc37. According to Buontempo et al., the inhibition of Cdc37 phosphorylation by CX-4945 leads to an impairment of the BiP/Hsp90/Cdc37 complex and thus to the downregulation of GRP78 (BiP) [25]. This results in the upregulation of the UPR member proteins. Synthetic oleanane triterpenoids (SOTs) are a novel class of GRP78-targeting agents. A representative of this class is 1-[2-cyano-3,12-dioxooleana-1,9(11)-dien-28-oyl]-4(-pyridin-2-yl)-1H-imidazole (2P-Im), which can be combined with ixazomib for the treatment of MM cells [26]. Another agent that directly inhibits GRP78 is HA15. Its combination with bortezomib showed synergistic effect on MM cell lines NCI–H929 and U266 cells [27].

The last member of this functional category is YUM70. Combined with the topoisomerase inhibitor topotecan or the HDAC inhibitor vorinostat, YUM70 expresses a synergistic antitumor effect against the cancer cell lines PANC-1 and UM59 [28]. While topotecan and vorinostat act as reactive oxygen species (ROS) and ER Stress inducers, YUM70 inhibits GRP78, hence exacerbating the accumulation of misfolded proteins and hyperactivating the UPR.

### 4.2. Therapies Using UPR Pathway Inhibitors

This category groups therapies that directly influence members of each of the three branches of the UPR. They are depicted in orange in Figure 3 and are detailed systematically in Table 2.

Vandewynckel et al. explore the synergism of oprozomib with agents that inhibit different members of the UPR pathway. Salubrinal, a substance that blocks eIF2α dephosphorylation, showed the most promising result with the greatest reduction in cell viability levels. Nelfinavir, an HIV protease inhibitor, also has ER Stress-related effects. The antitumor effects of nelfinavir lie in its ability to inhibit the site-2-protease (S2P), thereby causing accumulation of uncleaved ATF6 and stalling this branch of the pathway. It is worth mentioning that nelfinavir also inhibits the ABCB1 (P-gp) pump and the nuclear factor erythroid 2-related factor 1 (Nrf1/NFE2L1). While ABCB1 is widely known as an efflux pump, Nrf1/NFE2L1 is responsible for upregulating proteasome expression (Nrf1/NFE2L1 should not be confused with Nuclear respiratory factor 1, NRF1). In addition, Vandewynckel et al. explored the synergism of oprozomib together with the second-generation PERK-inhibitor GSK2656157 and the first-generation IRE1α-inhibitor 4μ8C [29]. Similarly, Fujimoto et al. and Zhang et al. explored the use of the first-generation PERK-inhibitor GSK2606414 in synergy with tunicamycin/cisplatin and digoxin, respectively [30,31]. Another strategy using nelfinavir was described by Abt et al. [32]. They identified a strong synergistic effect of the classic PIs bortezomib or carfilzomib and nelfinavir against the cancer cell lines Caki-2, A498 and MZ1774. Inhibition of the IRE1α pathway was approached by Sheng et al. and Chien et al. They used different inhibitors of the RNase activity of IRE1α, namely MKC8866 + Cabazitaxel, STF-083010/2-Hydroxy-1-naphthaldehyde, and 3-Ethoxy-5,6-dibromosalicylaldehyde/toyocamycin + bortezomib respectively [33,34].

Another approach investigates the synergy between Compound C, also known as dorsomorphin, and 2-DG/tunicamycin. According to the results of Saito et al., Compound C, in combination with 2-DG, inhibits the transcription of both GRP78 and other members of the UPR pathway. Therefore, instead of hyperactivating the UPR, the latter is downregulated. The target cells can no longer cope with the ER Stress and therefore succumb of cytotoxicity [35]. Additionally, Saito et al. established that Compound C has a different mode of action compared to other pan-UPR inhibitors. Therefore, they continued their investigation further to discover that the combination of Compound C and phenformin under 2-DG stress conditions resulted in even higher levels of cytotoxicity, specifically in the 786-O cell line. Another strategy in this category involves a lead compound, termed #17, in synergy with 2-DG. The treatment was tested on different cancer cell lines from solid tumors such as HeLa, A549, H1299, and HCT116 [36]. Huang et al. discovered experimentally that compound #17 downregulates the expression of the UPR members GRP78, CHOP, and ATF4 both in the presence of 2-DG and tunicamycin. The synergistic treatment causes G2/M cell cycle arrest and consecutive apoptosis in the target cells. Although the exact chain of events causing apoptosis is not established, it was indicated that GRP78 overexpression and activation of the IRE1–XBP1s pathway may reverse the cell cycle arrest, induced by compound #17.

### 4.3. Therapies Using Signal Pathway Inhibitors

**Figure 4:**
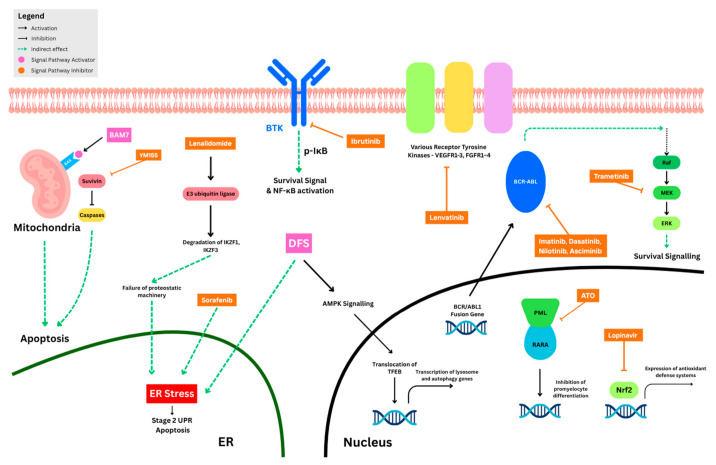
Signal pathway inhibitors and signal pathway activators. The diagram illustrates therapies targeting cellular signaling to exacerbate ER Stress. It shows how signal pathway inhibitors (in orange) disrupt survival signaling to overwhelm the ER. It also details how signal pathway activators (in pink) promote ER Stress or initiate pro-apoptotic cascades.

This group contains a heterogenous collection of molecules that indirectly cause an increase in ER Stress. They are highlighted in orange in Figure 4 and are summarized in Table 3.

Ibrutinib is a Bruton’s tyrosine kinase (BTK) inhibitor. Bruton’s kinase is a key enzyme in the B-cell receptor (BCR) signaling pathway. The latter is chronically hyperactivated in B-cell malignancies and serves as a major contributor to cell survival and proliferation. Inhibition of BTK results in loss of survival signaling. Ibrutinib may be combined with the glycosylation inhibitor 2-DG for the treatment of the activated B-cell-like (ABC) subtype of diffuse large B-cell lymphoma (DLBCL) [37]. However, resistance mechanisms have been indicated in DLBCL which are at least partially due to the UPR suppression, particularly the downregulation of XBP1. It was proved that the overexpression of XBP1s via adenoviral transduction potentiated the effect of ibrutinib against the ibrutinib-resistant ABC cell line OCI-ly10-IR. Finally, potential synergy was observed between the glycosylation inhibitor 2-DG—which induces pan-activation of the UPR and XBP1 upregulation —and ibrutinib, resulting in significantly enhanced reduction in cell viability in the OCI-ly10-IR cell line. Additionally, ibrutinib synergizes with the β2-selective proteasome inhibitor LU-102 [38]. Except for proteasome inhibition, LU-102 was observed to cause a decrease in cellular p-IκB levels, which in turn suppresses NF-κB activation. Ibrutinib treatment alone was also proven to cause a decrease in p-IκB and therefore diminish survival signaling. NF-κB activation is one of the main targets of the UPR transcription program and its synergistic inhibition by LU-102 and ibrutinib was shown to cause great decreases in tumor cell viability. Lastly, Ovejero et al. also explored the potential of ibrutinib, this time in combination with the ferroptosis inducer Ironomycin, against six MCL cell lines (JEKO1, JVM2, MAVER1, MINO, REC1, GRANTA519) [39]. Ironomycin causes mitochondrial dysfunction and ROS accumulation, thus indirectly increasing ER Stress and upregulating UPR. The two drugs expressed synergistic effects against MCL cell lines. Additionally, a downregulation of the BCR pathway was also observed.

Another approach involves the use of the multi-kinase inhibitor sorafenib in combination with the PIs ALLN (acetyl–leucyl–leucyl–norleucinal) and epoxomicin for the treatment of hepatoma cells. Honma et al. reported that treatment with sorafenib alone causes an increase in CHOP in the Huh7 and Hep3B hepatocellular carcinoma cell lines, but not in the highly differentiated immortalized human hepatocyte cell line OUMS29 [40]. Sorafenib is also indicated to be a potent ER Stress inducer and UPR activator [41]. In contrast, sorafenib, when combined with PIs, caused an accumulation of ubiquitinated proteins and downregulated the UPR members CHOP and XBP1. This synergistic treatment seems to cause substantial cytotoxicity by debilitating the cell’s mechanism to cope with the ER Stress, therefore causing necrosis and apoptosis. Similarly, Fan et al. explored the synergistic combination of sorafenib with the antibiotic clofoctol against the prostate cancer cell lines PC-3, DU145, and LNCaP [42]. Clofoctol is known to increase ER Stress and leads to the activation of all UPR members. When combined, the two drugs achieved synergy with the most prominent effect being on the PC-3 cell line.

**Table 3:** Therapies using signal pathway inhibitors.

Primary Drug (ER-Stress Inducer)	Accompanying Drug (Signal Pathway Inhibitor)	Mechanism of Action of the Accompanying Drug	Cancer Type/Cancer Cell Line	Author/Ref. No.
Bortezomib/Carfilzomib	Lopinavir	HIV-Protease inhibitors; inhibit Nrf2 to block the cellular antioxidant response.	Renal cell carcinoma cells	Abt. et al. [32]
2-DG (Glycosylation inhibitor)	Ibrutinib	BTK inhibitor; increases transcription of UPR genes (XBP1).	Diffuse large B-cell lymphoma cell lines	Zhang et al. [37]
LU-102 (PI)	Ibrutinib	BTK inhibitor; decreases p-IκB levels, suppressing NF-κB activation.	MM cells	Kraus et al. [38]
Ironomycin	Ibrutinib	BTK inhibitor; causes downregulation of the BCR survival pathway.	Mantle cell lymphoma cell lines	Ovejero et al. [39]
ALLN/Epoxomicin	Sorafenib	Multi-kinase inhibitor; potent ER-stress inducer and UPR activator.	Hepatocellular carcinoma cell lines	Honma et al. [40]
Clofoctol	Sorafenib	Multi-kinase inhibitor; potent ER Stress inducer and UPR activator.	Prostate cancer cell lines	Fan et al. [42]
Thapsigargin/Oligomycin	Imatinib, Dasatinib, Nilotinib, Asciminib	BCR:ABL1 inhibitors (TKIs) that synergize with metabolic inhibitors.	Chronic myeloid leukemia cell lines	Häselbarth et al. [43]
Antrocinol	Lenvatinib	Downregulates the expression of ATG5—a key regulatory molecule related to autophagy	Hepatocellular carcinoma cell lines	Lai et al. [44]
Onalespib (HSP90i)	Trametinib/MTA (Pemetrexed)	MEK inhibitor/Antifolate; targets resistance in KRAS-mutant cells.	Lung cancer Cell lines)	Yang et al. [45]
Bortezomib (PI)	Arsenic trioxide (ATO)	Binds PML/PML-RARA proteins; leads to autophagy-mediated degradation.	Acute Promyelocytic Leukemia cell lines	Ganesan et al. [46]
Romidepsin (HDACi)	Lenalidomide	Pleiotropic; activates E3 ubiquitin ligase to degrade IKZF1/3.	T-Cell Lymphoma cell line, Anaplastic Large Cell Lymphoma cell line	Cosenza et al. [47]
CuET (p97 inhibitor)	YM 155	Survivin inhibitor; prevents physical inhibition of caspases.	Prostate cancer cells	Majera et al. [48]

A few more therapies rely on combination treatments involving tyrosine kinase inhibitors (TKIs). Häselbarth et al. explored a different energetically-oriented approach for the induction of ER Stress [43]. As Ca^2+^ levels in the ER have a critical role in the maintenance of the ER proteostasis with low calcium levels causing the generation of ER Stress, inhibition of the Ca^2+^ ATP-ase serves as a possible therapeutic approach. The team explored the inhibition of the Ca^2+^ ATP-ase via a direct inhibitor—thapsigargin—in combination with the OXPHOS inhibitor oligomycin. Furthermore, they explored the synergy between these two metabolic inhibitors and different TKIs—Imatinib, Dasatinib, Nilotinib and Asciminib. They treated several myeloid leukemia cell lines, including the CML BCR::ABL1-positive cell lines K562, BV173, and KU812. The AML cell line HL60, which was used as a BCR::ABL1-negative control. Significant synergistical effects were discovered in some of the treated cell lines. Another TKI, Lenvatinib, may be combined with Antrocinol. Antrocinol is known to downregulate the expression of ATG5—a key regulatory molecule related to autophagy. Its suppression leads to stalling in the autophagy machinery. Lai et al. proved the synergistic effect of Antrocinol in combination with the TKI Lenvatinib on Lenvatinib-resistant Huh-7 and HepG_2_ cell lines (Huh-7/LR, HepG2/LR) [44]. They demonstrated that the combination of Lenvatinib and Antrocinol managed to reactivate the UPR in the Lenvatinib-resistant cell lines and induced apoptosis.

Yang et al. explored a novel approach for the treatment of drug-resistant KRAS-mutant lung cancer using an HSP90 inhibitor—onalespib—in synergism with either the MEK-inhibitor trametinib or the multitargeted antifolate (MTA) pemetrexed [45]. They established that the Epithelial-to-Mesenchymal Transition (EMT), observed in KRAS-mutant cells, serves a key role in the development of drug resistance against MEK inhibitors. Additionally, they discovered that this resistance is mediated by the cytosolic HSP90 with drug-resistant cancer cells exhibiting a relative overexpression of HSP90 compared to the non-drug-resistant variants. HSP90-mediated drug resistance was proven to be associated with the hyperactivation of the AXL/eIF4E pathway. Additionally, the AXL/eIF4E hyperactivation triggered a stress-induced stage 1 UPR with an increase in ER chaperones and a repression of the pro-apoptotic eIF2α and CHOP. Following this logic, inhibition of HSP90 with onalespib resulted in prominent hyperactivation of UPR genes and shifted UPR from its pro-survival PERK/JNK/ATF2-dependent state to its pro-apoptotic state, executed by the eIF2α/CHOP axis. Finally, a combination therapy using onalespib with trametinib or MTA (pemetrexed) showed high levels of synergy against the KRAS-mutant A549, H358 and the murine KP lung cancer cell lines, and logically, no synergy against the *KRAS* wild-type H3122 and PC9 lung cancer cells.

Ganesan et al. demonstrate the synergy between the proteasome inhibitor bortezomib and Arsenic trioxide (ATO) for the treatment of ATO-sensitive and RT-resistant acute promyelocytic leukemia (APL) [46]. APL is considered the most curable of all adult leukemias. In some cases, however, the NF-κB pathway—mediated ATO-resistance is observed. ATO is known to exert its action by binding to the PML and the PML-RARA proteins. This in turn leads to SUMOylation and subsequent polyubiquitination and proteasomal degradation of the PML portion. In this line of thought, the concurrent use of any proteasome inhibitor, bortezomib included, should prove antagonistic. It was discovered that in the case of combination treatment PML is degraded in proteasome-independent manner by autophagy. Additionally, bortezomib inhibited the stromal culture-induced activation of the NF-κB pathway, which is known to contribute to ATO-resistance when the primary blasts from APL are co-cultured with stromal cells. The combination also significantly induced ROS generation and decreased the mitochondrial membrane potentials of NB4 cells.

Cosenza et al. examined the potential synergy between romidepsin—a class 1 HDAC inhibitor and lenalidomide against the T-cell lymphoma cell lines Hut-78 and Karpas-299 [47]. Apart from their classic effects, histone deacetylases (class 2 HDAC) are responsible for keeping chaperones like HSP90 non-acetylated and active. Inhibition of class 2 HDACs requires much higher levels of romidepsin but eventually it has ER stress-induction effects as the deacetylation of HSP90 becomes inhibited. Lenalidomide has multimodal anticancer effects, with one of its main mechanisms of action involving the modulation of the E3 ubiquitin ligase complex—more specifically, its binding to a protein called Cereblon. This interaction alters substrate specificity and leads to the ubiquitination and proteasomal degradation of the transcription factors IKZF1 and IKZF3. The loss of these factors suppresses pro-survival signaling and results in downregulation of key pathways, including MYC and IRF4, as well as effects on angiogenesis and cytokine production. Ultimately, these changes lead to growth arrest and apoptosis in tumor cells.

Majera et al. combined the p97-dependent protein degradation pathway inhibitor CuET with the survivin inhibitor YM 155 for the treatment of the prostate cancer cell lines DU145 and PC3 [48]. While the ultimate effect of CuET on cancer cell lines is to inhibit the ERAD protein degradation machinery, YM 155 influences apoptosis. Survivin, as the name implies, is a crucial member of the IAP (Inhibitor of Apoptosis) family of proteins. Unlike some other IAPs, it does not directly inhibit caspases through physical binding. Instead, it regulates apoptosis indirectly by interacting with other proteins involved in caspase activation. The inhibition of survivin leads to increased caspase activation and promotion of apoptosis. CuET and YM 155 together exert a synergistic cytotoxic effect against the aforementioned cell lines.

In addition to the treatment using Nelfinavir, Abt. et al. explore the combination of bortezomib or carfilzomib and the HIV-protease inhibitor Lopinavir [32]. The mechanism of synergy lies in the ability of Lopinavir to downregulate nuclear factor erythroid 2-related factor 2 (Nrf2) [49]. Nrf2 is a transcription factor that acts as a master regulator of cellular antioxidant defense and homeostasis. Similarly to Nelfinavir, Lopinavir also inhibits the ABCB1 efflux pump.

Another molecular target is the Fms-like tyrosine kinase 3 (FLT3). FLT3 with internal tandem duplications (ITDs)—FLT3ITD—is a major oncoprotein in acute myeloid leukemia. The FLT3ITD-selective inhibitor AC220 (quizartinib) was tested in synergy with bortezomib against AML cell lines [50].

The epidermal growth factor receptor (EGFR) tyrosine kinase receptor inhibitor Erlotinib may be combined with ixazomib against solid tumors [51]. The same agent—erlotinib—was tested in combination with bortezomib against non-small cell lung cancer cell lines [52]. Vactosertib is a selective ALK5 inhibitor (TGF-β receptor type 1 inhibitor). It was examined in synergy together with bortezomib/ixazomib for the treatment of PI-resistant MM [53].

### 4.4. Signal Pathway Activators

The therapies in this category are illustrated in pink in Figure 4.

Combination Organelle Mitochondrial Endoplasmic Reticulum Therapy (COMET) is a therapeutic approach used by Milane et al. for the treatment of multidrug-resistant (MDR) triple-negative breast cancer (TNBC) on cell lines MDA-MB-231 and BT-549 [54]. Mitochondrial Network Disrupting Nanoparticles (MiNDs) loaded with three components—an anti-MFN2 peptide, the glycosylation inhibitor tunicamycin, and Bam7, which is a specific activator of the pro-apoptotic Bax—were applied. This therapeutic approach aims at disturbing the increased mitochondrial networks that are characteristic of MDR TNBC cells. Hypoxia significantly increases the number of mitochondrial networks. The anti-MFN2 peptide intuitively inhibits MFN2 and thus suppresses mitochondrial fusion, which decreases the number of pro-apoptotic binding sites on the outer mitochondrial membranes. The team proved that this strategy successfully managed to decrease the apoptotic threshold of the targeted cancer cells. Tunicamycin, the second component, serves to increase the ER Stress and to induce the UPR. Bam7 alone, being a direct activator of apoptosis, fails in significantly elevating the caspase 3 levels, while COMET treatment resulted in a multiple-fold increase in caspase 3 under both normoxic and hypoxic conditions.

Natural phytochemicals are being tested as possible anticancer drugs because of the absence of high cytotoxicity common with conventional chemotherapeutics. One such compound is a type of lignan—DFS ((−)-(2R, 3R)-1,4-O-diferuloylsecoisolariciresinol). Kwon et al. examined the synergy of DFS in combination with the autophagy inhibitor 3-methyladenine (3-MA) [55]. They treated different cancer cell lines, including the prostate cancer cell line DU145 and the colorectal cancer cell line SW480. It was established that DFS serves a dual role by both stimulating the generation of ER Stress and subsequent UPR activation. Additionally, itsimultaneously induces autophagy by activating AMPK signaling and subsequent nuclear translocation of TFEB. As autophagy serves as a pro-survival function against ER Stress, the team tested the possible synergy upon addition of an autophagy inhibitor, 3-MA. The combination resulted in synergy as 3-MA prominently increased cell death compared to the DFS-only approach.

### 4.5. Therapies Using Proteostasis Inhibitors

**Figure 5:**
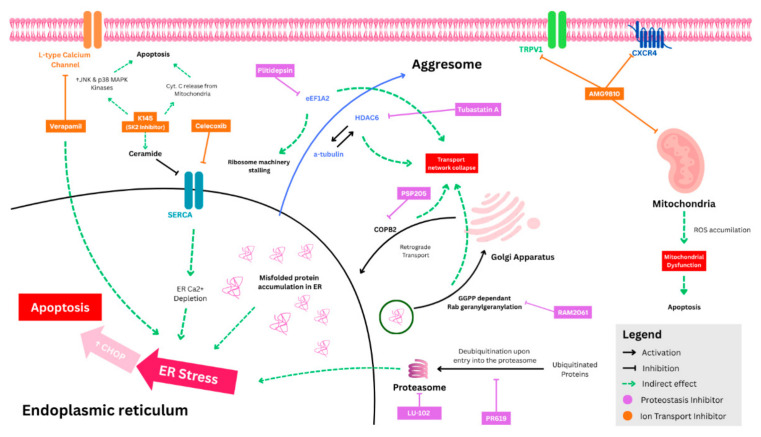
Proteostasis inhibitors and ion transport inhibitors. The diagram illustrates the therapies targeting the ionic environment of the cell and the processing of proteins. Proteostasis inhibitors (LU-102 and PR-619) are shown to block the clearance of misfolded proteins, leading to ER Stress. Simultaneously, ion transport inhibitors (including SERCA inhibitors like Celecoxib and Ca^2+^ channel blockers like Verapamil) disrupt the ER’s calcium balance. Together, these approaches disorder cancer cell homeostasis and shift the UPR from a pro-survival stage 1 to a pro-apoptotic stage 2.

This category includes therapeutic strategies that directly disrupt cellular proteostasis. They are highlighted in purple in Figure 5 and are visualized systematically in Table 4.

A possible approach is to combine two PIs as a strategy to overcomePI-resistance. Kraus et al. evaluated the synergy between the novel β2-selective proteasome inhibitor LU-102 and the classic PIs bortezomib and carfilzomib for the treatment of the cell lines RPMI8226, LP-1, AMO-1, U266, MM1S and MM1R [56]. The effect was a significant decrease in cell viability, as well as prolongation of the proteasome-inhibition effect compared to bortezomib/carfilzomib treatment alone. Instead of inhibiting the proteasome active site, it is possible to restrict the entry of ubiquitin-tagged proteins into the proteasome. Cai et al. examined the potential of a nanoparticle system, containing a tumor-homing peptide tLyP-1, Fe_3_O_4_ as a source ROS, ER Stress inducers, and PR-619, a pan-deubiquitinating enzyme (DUB) inhibitor [57]. Intuitively, as a pan-DUB inhibitor, PR-619 should stimulate protein degradation by shutting off the “rescue” deubiquitinase enzymes. However, DUBs are also required for the final stages of ERAD, where the ubiquitin molecules need to be trimmed from the misfolded proteins prior to entry into the proteasome. Consequently, PR-619 inhibits ERAD and protein degradation, hence causing accumulation of ER Stress. Together with Fe_3_O_4_ in the form of a nanoparticle, they show a synergistic antitumor effect against cancer cell lines 786-O, HK-2, and PC-3.

Another strategy is to inhibit the various processes of vesicular trafficking that have a key role in proteostasis. Haney et al. explore the synergy between bortezomib and geranylgeranyl diphosphate synthase inhibitor (GGDSI) RAM2061 for the treatment of multiple myeloma cell lines [58]. GGDSIs cause a reduction in levels of geranylgeranyl pyrophosphate (GGPP), which in turn are needed for Rab GTPase geranylgeranylation. The inhibition of this process causes collapse in the vesicular trafficking at the very beginning of the protein lifecycle. The consequent protein accumulation leads to ER Stress and upregulation of the UPR. Previous studies have established that the UPR upregulation by GGDSI is GGPP-dependent [59]. Apart from anterograde transport, it is possible to inhibit retrograde transport too. PSP 205 is a novel phenyl sulfonyl piperidine that synergizes with the proteasome inhibitor (MG132) against the cancer cell line HT29 [60]. The mechanism of action of PSP205 in cancer cell apoptosis is believed to be caused by its inhibitory effect on COPB2. COPB2 is a critical subunit of the coatomer protein (COPI), which is responsible for retrograde transport from the Golgi complex to the ER. Inhibition of COPB2 results in disruption of cellular transport and a literal “traffic jam” in protein trafficking, which results into accumulation of proteins and subsequent ER Stress. Another approach is to impede the transport of ubiquitin-tagged proteins to the aggresome. Temozolomide and Tubastatin A synergize against the glioblastoma cell lines U343, U373, U138, LNZ308, A172, U118, U251 and U87 [61]. While TMZ acts as an indirect ER Stress inducer by damaging DNA, Tubastatin A has a specific HDAC6-targeting mechanism. HDAC6 facilitates dynein-mediated transport of ubiquitinated misfolded proteins to aggresomes by serving as a scaffold linking cargo to the motor complex. Consequently, it is responsible for transporting the misfolded proteins from the ER via the microtubules to the aggresome [62]. Additionally, HDAC6 is responsible for deacetylating HSP90, which keeps the chaperone in active state [63]. Therefore, inhibition of HDAC6 causes a collapse in this transport network and ultimately worsens ER Stress.

Another potential agent is plitidepsin, a cyclic depsipeptide that has a wide range of anticancer activities. Losada et al. examined the synergy of plitidepsin and the classic PI bortezomib for the treatment of mouse-xenografted RPMI-8226 cells [64]. Plitidepsin exerts its anticancer effect by inhibiting eukaryotic translation elongation factor 1 Alpha 2 (eEF1A2), which serves a dual role. It is involved in translation but also acts as a molecular chaperone and a transport factor by facilitating the trafficking of misfolded proteins to the aggresome. Its overall effects are to cause an increase in ER Stress, ROS generation, and inhibition of protein degradation, accompanied by paradoxical stimulation of CHOP degradation and inhibition of autophagy.

**Table 4:** Therapies using proteostasis inhibitors.

Primary Drug (ER-Stress Inducer)	Accompanying Drug (Proteostasis Inhibitor)	Mechanism of Action of the Accompanying Drug	Cancer Type/Cancer Cell Line	Author/Ref. No.
Bortezomib/Carfilzomib	LU-102	Beta2-selective proteasome inhibitor; prolongs the duration of proteasome inhibition	MM cell lines	Kraus et al. [56]
Fe_3_O_2_ (Nanoparticle)	PR-619	Pan-deubiquitinating enzyme (DUB) inhibitor; inhibits ERAD by preventing ubiquitin trimming required for proteasomal entry	Renal cell adenocarcinoma and prostate cancer cell lines	Cai et al. [57]
Bortezomib	RAM2061	Reduces GGPP levels, inhibits Rab GTPase geranylgeranylation.	MM cell lines	Haney et al. [58]
MG132 (Proteasome Inhibitor)	PSP 205	Phenyl sulfonyl piperidine that inhibits COPB2; disrupts Golgi-to-ER retrograde transport, causing a protein “traffic jam”	Colon cancer cell lines	Samanta et al. [60]
Temozolomide (TMZ)	Tubastatin A	Specific HDAC6 inhibitor; disrupts the microtubule-dependent transport of misfolded proteins from the ER to the aggresome	Glioblastoma multiforme cell lines	Li et al. [61]
Bortezomib	Plitidepsin	Cyclic depsipeptide (protein synthesis inhibitor); causes ER Stress via ROS generation and inhibition of protein degradation/autophagy	MM cell lines, breast cancer and cervical cancer cell lines	Losada et al. [64]

### 4.6. Therapies Using Ion Transport Inhibitors

Ion channels represent a potential therapeutic target in oncology. Because intracellular ion concentrations heavily influence proteostasis and protein folding, the targeted disruption of ion homeostasis offers a potential therapeutic strategy. The following therapies addressing these mechanisms are highlighted in orange in Figure 5 and are summarized in Table 5.

Wallington-Beddoe et al. investigated the ER Stress-generating capabilities of sphingosine kinase 2 inhibitors K145 and ABC294640 in synergy with bortezomib for the treatment of myeloma cell lines [65]. Inhibition of the SK2 pathway causes a shift in the ceramide/sphingosine-1-phosphate rheostat towards the accumulation of ceramide. Classically, ceramide is known to cause apoptosis by inserting itself in the mitochondrial membrane and facilitating the release of the pro-apoptotic cytochrome c. Additionally, it has been established that ceramide can directly cause ER Stress by inhibiting the expression of the sarcoplasmic/endoplasmic reticulum Ca^2+^-ATPase (SERCA) [66]. In this way, ceramide disturbs the calcium homeostasis in the ER and causes UPR upregulation. Additionally, ceramide activates the pro-apoptotic JNK and p38-MAPK kinases. Another therapy that targets SERCA was developed by Cusimano et al. [67]. They demonstrated the synergy between the proteasome inhibitor MG132 and the selective COX-2 inhibitor celecoxib against human liver tumor cells. Apart from its classical function, celecoxib is known to exhibit anti-proliferative properties against cancer cell lines. It has been established that celecoxib is an inhibitor of SERCA [68]. Inhibition of SERCA and the consequent depletion of Ca^2+^ in the ER lead to the generation of severe ER Stress.

It is also possible to target the plasma membrane ion channels. One of these is the Transient Receptor Potential Vanilloid type 1 (TRPV1) calcium channel. A synergistic effect exists between the TRPV1 inhibitor AMG9810 and the proteasome inhibitors bortezomib and carfilzomib for the treatment of MM cell lines [69]. The application of AMG9810 alone on MM cells suppresses their viability. The mechanism of action involves Ca^2+^ flux in mitochondria and subsequent accumulation of ROS, as well as suppression of the CXCR4 expression in tumor cells. Additionally, AMG9810 and bortezomib exert a synergistic effect in suppressing the MM cell viability. bortezomib is known to induce ER Stress and thus compensatory ubiquitin pathway upregulation. Treatment with AMG9810, however, reversed this effect by inhibiting the ubiquitin pathway, which could explain the observed synergy. Furthermore, AMG9810 inhibits the bortezomib-induced upregulation of HSP70, which adds to the synergistic effect. Alternatively, Meister et al. explored another potential synergistic therapy by combining verapamil, a classic Ca^2+^ channel inhibitor used for treatment of arrhythmias and hypertension, and the classic PI bortezomib [70] on RPMI 8226, ARH 77 and JK-6L cells. Bortezomib acts directly as an ER Stress inducer. Verapamil treatment alone does not significantly alter the viability of the treated cancer cells. However, it was proven that it caused an increase in the expression of PERK and ATF4. Upon combination treatment, however, an increase in the UPR activation, NF-κB inhibition, ROS production, and misfolded protein accumulation was observed.

**Table 5:** Therapies using ion transport inhibitors.

Primary Drug (ER-Stress Inducer)	Accompanying Drug (Ion Transport Inhibitor)	Mechanism of Action of the Accompanying Drug	Cancer Type/Cancer Cell Line	Author/Ref. No.
Bortezomib	K145 (SK2 Inhibitor)	(1) Accumulates ceramide (by lowering S1P) (2) Inhibits SERCA expression and disturbs ER calcium homeostasis.	MM cell lines	Wallington-Beddoe et al. [65]
MG132	Celecoxib (COX-2 Inhibitor)	Direct inhibitor of SERCA, leading to the depletion of Ca^2+^ in the ER.	Human liver tumors	Cusimano et al. [67]
Bortezomib/Carfilzomib	AMG9810	Inhibits TRPV1 channels, causing mitochondrial Ca^2+^ flux, ROS accumulation, and inhibition of the ubiquitin pathway.	Breast cancer cell lines	Beider et al. [68]
Bortezomib	Verapamil	Ca^2+^ channel inhibitor; increases expression of PERK and ATF4 to enhance UPR activation.	MM cell lines	Meister et al. [70]

### 4.7. Conventional Chemotherapeutics

Evangelisti et al. studied the synergy of the sphingosine kinase 1 and sphingosine kinase 2 inhibitor SKi (2-(p-hydroxyanilino)-4-(p-chlorophenyl)thiazole) and vincristine against the T-ALL cell lines Molt-4 and Jurkat [71]. They discovered a strong potentiation of ER Stress and related UPR activation in the Jurkat cell line in response to treatment with SKi. Additionally, the team established that the autophagy inhibitor chloroquine, when combined with SKi, resulted in evident reduction in cell viability, thus proving that UPR activation and autophagy are crucial to maintaining the homeostasis in the treated cell lines.

Three of the examined therapeutic approaches utilize synergistic strategies involving cisplatin. Erzurumlu et al. evaluated the synergy between Astaxanthin and the conventional chemotherapeutic cisplatin against the cancer cell line LNCaP [72]. Astaxanthin belongs to the xanthophyll family of compounds. Upon treatment with Astaxanthin an increase in ER Stress and autophagy was observed. Another natural compound with ER Stress-inducing capabilities is xanthohumol (XN). Vecchio et al. demonstrate the synergy of XN in combination with cisplatin against the cancer cell line UMSCC-103 [73]. XN proved to increase ER Stress and therefore UPR activation. Another study attributed the ER Stress-causing effect of XNto proteasome inhibition [19]. Finally, cisplatin may be combined with the GFAT inhibitor Diazo-5-oxo-L-norleucine (DON) [74]. A synergistic cytotoxic effect against the cancer cell lines Calu-3 and H2009 was reported.

### 4.8. Others

An alternative way to inhibit the metabolism of cancer cells has been proposed by Fan et al. [75]. They investigated the synergy between the glycosylation inhibitor 2-DG and Berberine against the cancer cell lines A549 and H460. Apart from the classic ER Stress-inducing effect, 2-DG also mimics a glucose deprivation state in the cells. Additionally, Berberine inhibits complex I of OXPHOS and exhibits AMPK-activation effects and subsequent mTOR-suppression effects. This combination of energy deprivation and accumulation of misfolded proteins ultimately leads to cell death.

Another unconventional approach is the combination treatment using the disulfiram (DSF)-derivative CuET in synergy with the JMJD3/UTX inhibitor GSK J4 against the esophageal squamous cell cancer cell lines TE10 and KYSE410 [76]. Ditiocarb (DTC), the active metabolite of DSF, is known to complex with Cu and form the chelate complex bis(diethyldithiocarbamate)-copper (CuET). CuET preferentially accumulates in tumor cells and is known to inhibit the p97-dependent protein degradation pathway, thus leading to the accumulation of proteins and ER stress. GSK J4 is an inhibitor of the histone demethylases JMJD3 and UTX. Its crucial effect is to facilitate the silencing of oncogenes. GSK J4 and CuET together exert synergistic cytotoxicity against the aforementioned cancer cell lines.

## 5. Discussion

Synergistic therapies targeting specific cellular mechanisms are currently emerging as a new perspective for cancer treatment. They allow for the simultaneous targeting of multiple elements of cellular homeostasis and proteostasis. They rely on the differences between the metabolic states of normal and cancer cells and exploit them to target the latter with higher levels of precision.

Naturally, within the presented classification framework, the widest variety is observed among the therapies aiming to specifically inhibit signaling pathways inside the cell. Almost all listed agents are kinase inhibitors. Most of the compounds are FDA-approved. This is expected, considering the vast number of molecules that could be targeted and the even wider range of downstream effectors that may be influenced. Consequently, signal pathway inhibitors hold the greatest promise for successful clinical translation.

From mechanistical point of view, the architecture of the UPR and its relation to ER Stress and the proteasome creates a suitable environment for vertical inhibition. While ER Stress inducers act on upstream elements such as proteasome, UPR feedback inhibitors that specifically target the chaperone GRP78, prove to be an inseparable part of future cancer treatment, considering their role in preventing the core feedback inhibition loop of the UPR. Eliminating that mechanism directly inactivates the major regulatory switch that prevents the UPR from shifting from its pro-survival to its pro-apoptotic mode. Initially, this approach seems to be the most logical as it directly prevents the cells from downregulating the UPR. However, some of these compounds are known to exhibit toxicity. EGCG, a green tea extract, which was used by two of the mentioned studies, may cause toxicity in higher doses [77]. Numerous clinical trials evaluating the therapeutic potential of EGCG are registered on ClinicalTrials.gov [78]. HNK is another natural compound that is extracted by the bark of *Magnolia officinalis*. Despite some data form studies, HNK appears to be safe at typical supplemental doses [79]. SR9009, which is an experimental REV-ERB agonist that is involved in indirect downregulation of GRP78, may disrupt normal circadian rhythms. Finally, Moxe, which is FDA-approved for the treatment of hairy cell leukemia (HCL), is also related to some side effects, particularly Hemolytic Uremic Syndrome (HUS) and Capillary Leak Syndrome (CLS). A noteworthy agent is IT139, which was observed to suppress GRP78 at the transcriptional level [80]. Another agent is the ruthenium-based compound BOLD-100 [81], which is supposed to exert its effect by downregulating GRP78, with two clinical trials currently underway [82,83]. Overall, the group of UPR Feedback Inhibitors shows promising potential, stemming from its unique mechanism of action. There is still plenty of room for the development of better, more selective agents with lower systemic toxicity. Essentially, UPR is a signaling network purposed to trigger a gene transcription program. At base-level activity, its pro-survival function is materialized by an increase in the expression of proteasome, chaperones, ERAD components, proteases and other factors responsible for the maintenance of proteostasis. Intuitively, specific targeting of its signaling branches renders the pathway defective. The category of UPR Pathway Inhibitors includes drugs that rely on this mechanism. One of the most prominent agents in the group is the first-in-class PERK inhibitor GSK2606414. Despite its targeting potential and potent anticancer effect, GSK2606414 has been associated with pancreatic toxicity. It should be noted that GSK2606414 has also been studied for its effects on neurodegeneration due to influence on the UPR [84,85]. Another PERK inhibitor—HC-5404 [86] is also being investigated. It recently completed Phase 1 trial [87]. In addition to the listed agents, Ceapins which are a class of pyrazole amides, act as selective inhibitors of ATF6 [88]. The S2P inhibitor Nelfinavir has been tested in combination with bortezomib in 2 phase 1 clinical trials [89,90]. MKC8866 (also known as ORIN1001), an IRE1α inhibitor (inhibitor of the RNase domain), has completed Phase 2 as an anticancer agent in two clinical trials [91,92]. In summary, there is still a clinical gap in the group of UPR Pathway Inhibitors, as no FDA-approved agent that targets directly any of the members of the three UPR branches currently exists. Unlike cancer cells, normal cells do not experience such high levels of unregulated protein production and therefore do not have such high requirements for proteasomal degradation. Additionally, their UPR system is operating at negligible levels. Therefore, healthy cells seem to be naturally resistant to this therapeutic strategy, unlike cancer cells which are highly dependent on it and hence vulnerable. The agents that fall into the categories of Proteostasis Inhibitors and Ion Transport Inhibitors are also metabolically intertwined with the UPR. Both categories include drugs that impede normal intracellular trafficking and protein folding. When combined with a catalyst such as PIs or glycosylation inhibitors, they cause a literal collapse of the normal protein lifecycle. The drugs classified as proteostasis inhibitors should currently be viewed as preclinical research tools rather than clinical therapeutic agents. LU-102, an irreversible selective β2-selective PI, is an interesting agent, as the PIs used today primarily target the β5 subunit of the proteasome. However, it should be noted that the standalone efficacy of LU-102 is relatively low when compared to other PIs, which has limited its transition into the clinic. Tubastatin A, another preclinical tool, has now been replaced by other, newer agents that are being enrolled in clinical trials [93]. The combination of PIs and HDAC inhibitors is not by far an entirely new concept as many studies have explored their potential combination [94,95]. One noteworthy combination is that of bortezomib and panobinostat, which is approved for clinical use. Another combination that underwent Phase 1 and Phase 2 clinical trials is that of bortezomib and ricolinostat [96].

A comprehensive overview of protein metabolism and proteotoxicity was provided by Ho Zhi Guang et al. [16]. They outlined the main housekeeping mechanisms that ensure proteostasis—Ubiquitin Proteasome System (UPS), Macroautophagy (Autophagy-Lysosome System), Aggresome Pathway, Heat-Shock Protein (HSP) Chaperone System, Integrated Stress Response (ISR), Endoplasmic Stress (ER) and the Unfolded Protein Response (UPR). Indeed, most of the drugs used in the synergistic strategies explored in this review could be classified as acting on one of the aforementioned regulatory mechanisms. Most susceptible to such proteostasis-targeting treatments are those cancer types that experience high levels of proteotoxicity. Such cancer types are MM and MCL, for the treatment of which proteasome inhibitors are approved, as well as TNBC. Additionally, cancer with hyperactivated mTORC1 pathway also experience high levels of ER Stress [16]. These include renal cell carcinoma, breast cancer, glioblastoma and endometrial cancer. Therefore, synergistic therapies targeting the UPR might show exceptionally high potential against these cancer types. It should be noted that the combination of bortezomib specifically with some other drug classes has been explored in an older 2010 study [97]. The paper highlights the potential of combination therapies both against hematologic malignancies and solid tumors.

Despite their high precision, targeted therapies also suffer resistance as tumor cells adapt. For instance, MM cells develop resistance to bortezomib by upregulating proteasome components such as the β5 subunit (PSMB5), by introducing point mutations in the gene of the latter, the so-called PSMB5 gene [98]. Additionally, cancer cells activate alternative protein-degradation pathways such as the autophagy–lysosome pathway. It should be mentioned that, in contrast, the efflux pump (P-gp) that is involved in multidrug resistance (MDR) has lower contribution to overall resistance to therapy. Some of these escape mechanisms can be effectively addressed by combination therapies. Firstly, they allow for the simultaneous inhibition of parallel pathways which, in this case, are protein degradation pathways. Additionally, by applying them simultaneously, the probability of a cancer cell lineage developing specific resistance to both therapies significantly drops. Because those therapies that achieve vertical inhibition (primary ER Stress inducers and UPR feedback inhibitors) act together on a single pathway, they potentiate each other’s effects. Lastly, synergistic therapies achieve broader coverage of all subpopulations. All these advantages render synergistic therapies a valuable tool for cancer treatment, especially in the context of ER Stress-targeting therapies [99]. It may be summarized that synergistic therapies targeting the UPR are most potent against those cancer types characterized by high levels of protein metabolism such as secreting tissues and antibody-producing cells. This predisposition, however, also defines which organs and tissues will potentially suffer from side toxicity.

Recent research reveals that ER Stress is not merely just an intrinsic survival mechanism within the tumor microenvironment (TME); it also directly suppresses T-cell activity. The process is known as Transmissive ER Stress (TERS) [100] and is observable in the site of tumorigenesis [101], though it may also be involved in other pathologies such as the pathogenesis of neurodegenerative diseases [102]. When tumor cells undergo chronic ER Stress, they secrete a cocktail of proinflammatory and stress-inducing factors such as soluble damage-associated molecular patterns (DAMPs), as well as many other metabolites such as lactate, fatty acids, ROS, etc. They have a key role in “transmitting” the ER Stress to the surrounding immune cells. Specifically, activation of the PERK-eIF2α pathway in the TME has been shown to induce a metabolic shift in T-cells, leading to upregulation of inhibitory receptors like PD-1, which drives T-cell exhaustion [103]. Furthermore, ER Stress also promotes recruitment and activation of Myeloid-Derived Suppressor Cells (MDSCs), which release ROS that inhibit CD8+ cytotoxic T-cells. Apart from inhibiting endogenous immunity, TERS may decrease the efficacy of immunotherapy [1].

Activation of the UPR, especially the IRE1α–XBP1 axis, has been shown to enhance PD-L1 expression and drive immunosuppressive signaling within the tumor microenvironment, linking ER Stress to immune checkpoint regulation and tumor immune evasion [104].

A solid link exists between ferroptosis and ER Stress. As noted by Bhandary et al., the synergy between ferroptosis and the UPR is driven by a “vicious cycle” where ROS-induced oxidative stress directly interferes with the thiol–disulfide exchange and inhibits enzymes like PDIs [105]. This inhibition causes the accumulation of misfolded proteins and the subsequent activation of the UPR. This ER Stress causes calcium leakage from the ER into the mitochondria, leading to the ferroptosis, iron-mediated Fenton reactions, which further accelerate ROS production and lock the cell in a self-sustaining loop of oxidative damage and ER Stress.

While the synergistic strategies identified in this review demonstrate significant potential for overcoming chemoresistance, some limitations must be acknowledged. Most of the analyzed studies rely on in vitro cell line models and in vivo murine xenografts. While they are essential steps in the proof-of-concept, they do not fully replicate the complexity of the human TME, nor do they account for hypoxia, nutrient deprivation, and local and systemic immune responses. The UPR is a fundamental survival mechanism for highly secretory healthy cells, such as pancreatic acinar cells and hepatocytes. Systemic treatment with potent ER Stress inducers and UPR modulator agents may lead to off-target toxicities that are not yet fully characterized in clinical settings. Such reports exist throughout literature, regarding gastrointestinal and cardiovascular toxicity [106,107,108]. Tumor heterogeneity remains a significant barrier with different clones exhibiting varying degrees of ER Stress and UPR activation. Finally, this review is limited by the search parameters used in the included databases. Although we utilized a systematic approach, recent or niche experimental findings published in non-indexed journals or in languages other than English may have been excluded, potentially omitting emerging therapeutic compounds.

## 6. Materials and Methods

### 6.1. Study Design

**Figure 6:**
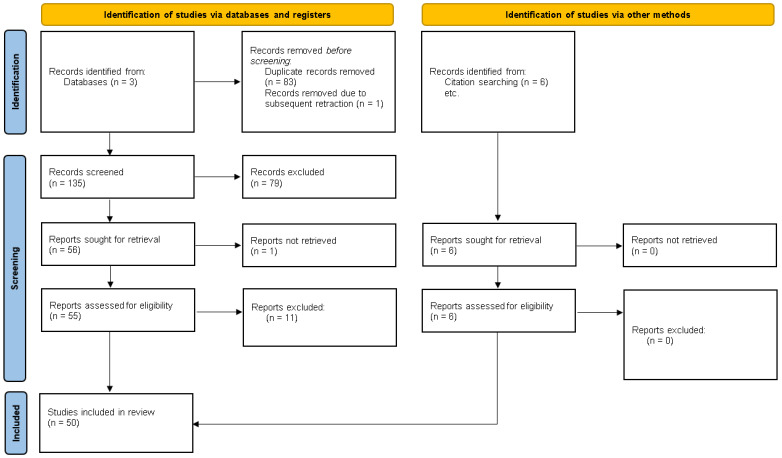
Identification and selection procedure of the articles in this review (adapted from PRISMA 2020 [109]).

This study was conducted as a systematic qualitative synthesis of the existing literature (Figure 6). The methodology followed the PRISMA (Preferred Reporting Items for Systematic Reviews and Meta-Analyses) guidelines and their extension for scoping reviews to ensure a transparent and reproducible selection process [109,110]. The analysis aimed to identify, evaluate, and categorize original research papers focused on UPR-mediated synergistic therapies in cancer.

### 6.2. Search Strategy

For the purposes of this review 3 databases were searched—PubMed, Scopus and Web of Science. Results were filtered for publications published until 12 February 2026. The search was additionally limited to English-language papers only. Two keywords were used in combination—“UPR” and “synergistic therapy”. This combination ensured that all papers related to the UPR and synergistic therapeutic approaches will be screened. A total of 219 results were identified.

### 6.3. Inclusion Criteria

The review focuses only on studies exploring a (1) direct or indirect manipulation of the UPR pathway for the treatment of different cancers. The publications include (2) synergistic therapeutic approaches with at least 1 of the therapeutic agents exhibiting an effect on the ER Stress and the UPR. The papers include (3) assays on cell viability and apoptosis.

### 6.4. Exclusion Criteria

Publications that combined a (1) genetic engineering method together with another drug (only if they do not explore an alternative non-genetic engineering synergistic approach) were excluded. Additionally, (2) publications that did not have data from cell viability/apoptosis assays were discarded. Publications that explored (3) therapeutic approaches that did not target the UPR were also not included.

### 6.5. Analysis

Out of 219 results, 83 were duplicates and 1 publication was subsequently retracted. This reduced the total number of papers to 135. Based on initial screening, only 56 were selected. Of those 56 studies, 1 could not be retrieved. Due to incoherence to our criteria 11 more publications were excluded. This led to a total of 44 articles. Another 6 additional papers were discovered via other methods. This led to a final total of 50 articles. It should be mentioned that within the presented classification, one of the publications described two different therapeutic approaches that belonged to two different functional categories. Relevant data from these included studies—including cancer cell lines used, pharmacological agents, and molecular outcomes—were extracted and managed using Microsoft Excel (Microsoft Corp, Redmond, WA, USA).

## 7. Conclusions

The UPR proves itself as a key regulatory and survival mechanism in cancer cells. While its activity is necessary for cancer cell survival, its hyperactivation proves successful in driving tumor cells into apoptosis. Monotherapies, as specific and targeted as they are, often prove incapable of fully shifting the UPR into its pro-apoptotic stage 2. Synergistic therapies overcome cancer adaptation and prove themselves as a promising therapeutic approach. The ER Stress level and UPR activation may be influenced via a huge variety of therapeutic agents that target a plethora of different cellular processes, which proves the key role of the UPR in cellular homeostasis. New agents are emerging as potential modulators of the UPR.

Moving forward, the success of UPR-targeted oncology will likely depend on the identification of biomarkers that predict ER Stress vulnerability, as well as the improvement of drug delivery systems to minimize off-target effects in healthy, proteostatically active tissues. Ultimately, leveraging the UPR not just as a marker of stress, but as a functional target for synergy, offers a powerful strategy to overcome long-standing chemoresistance in aggressive malignancies.

## Figures and Tables

**Table 1:** Therapies using UPR feedback inhibitors.

Primary Drug (ER-Stress Inducer)	Accompanying Drug (UPR Feedback Inhibitor)	Mechanism of Action of the Accompanying Drug	Cancer Type/Cancer Cell Line	Author/Ref. No.
2-DG (Glycolysis/Glycosylation inhibitor)	Moxe	Prevents the upregulation of GRP78, keeping UPR sensors perpetually active.	Xenograft mouse models of mantle cell lymphoma, Burkitt’s lymphoma, patient-derived acute lymphoblastic leukemia	Gsottberger et al. [20]
Plant based	HNK (Honokiol)	Binds to the ATP-ase domain of GRP78; prevents sequestration of UPR sensors	Neuroectodermal tumor cell lines, melanoma and glioblastoma cells	Martin et al. [21]
Quercetin	EGCG	Binds to the ATP-ase domain of GRP78; prevents sequestration of UPR sensors.	Breast cancer cells	Li et al. [22]
Taxol or Vinblastine	EGCG	Binds to the ATP-ase domain of GRP78; inhibits chaperone function.	Breast cancer cells	Wang et al. [23]
Bortezomib	SR9009	Activates REV-ERBα/β to downregulate GRP78 and reduce autophagy.	MM cell lines (RPMI8226 and U266) in vitro and in vivo nonobese diabetic/severe combined immunodeficient (NOD/SCID) murine xenograft MM model	Wang et al. [24]
Bortezomib	CX-4945 (CK2-inhibitor)	Inhibits Cdc37 phosphorylation, impairing the BIP/Hsp90/Cdc37 complex to downregulate GRP78.	Acute lymphoblastic leukemia patients	Buontempo et al. [25]
Ixazomib	2P-Im	Directly inhibits GRP78	MM cells and in vivo in a murine model of plasmacytoma	Luo et al. [26]
Bortezomib	HA15	Binds to the ATP-ase domain of GRP78; prevents sequestration of UPR sensors.	MM cells	Chen et al. [27]
Topotecan or Vorinostat	YUM-70	Binds to the ATP-ase domain of GRP78; prevents sequestration of UPR sensors.	Pancreatic cancer cell lines and in vivo in a pancreatic cancer xenograft model	Samanta et al. [28]

**Table 2:** Therapies using UPR pathway inhibitors.

Primary Drug (ER-Stress Inducer)	Accompanying Drug (UPR Pathway Inhibitor)	Mechanism of Action of the Accompanying Drug	Cancer Type/Cancer Cell Line	Author/Ref. No.
Oprozomib	Salubrinal	Inhibits eIF2α dephosphorylation	Hepatocellular carcinoma cell line and in vivo orthotopic and xenograft models	Vandewynckel et al. [29]
Oprozomib	Nelfinavir	Inhibits Site-2-Protease (S2P), causes ATF6 accumulation	Hepatocellular carcinoma cell line and in vivo orthotopic and xenograft models	Vandewynckel et al. [29]
Tunicamycin/Cisplatin	GSK2606414	First-generation PERK inhibitor	Human cervical squamous carcinoma cell line	Fujimoto et al. [30]
Digoxin	GSK2606414	First-generation PERK inhibitor	Cervical cancer cell line	Zhang et al. [31]
Bortezomib/Carfilzomib	Nelfinavir	Inhibits Site-2-Protease (S2P)	Clear cell renal cell cancer cell lines	Abt et al. [32]
Cabazitaxel	MKC8866	Inhibits IRE1alpha RNase activity	Prostate cancer cell lines	Sheng et al. [33]
Bortezomib	STF-083010	Inhibits IRE1alpha RNase activity	Pancreatic cancer cells	Chien et al. [34]
Phenformin (under 2-DG stress)	Compound C (Dorsomorphin)	Synergistic cytotoxicity via UPR downregulation	Human fibrosarcoma, renal cell carcinoma, cervical carcinoma cell lines	Saito et al. [35]
2-DG	Lead Compound #17	Downregulates GRP78, CHOP, and ATF4 expression	Cervical adenocarcinoma, non-small cell lung cancer, colorectal carcinoma cell lines	Huang et al. [36]

## Data Availability

No new data were created or analyzed in this study. Data sharing is not applicable to this article.

## Abbreviations

The following abbreviations are used in this manuscript:

2-DG	2-deoxy-D-glucose
3-MA	3-methyladenine
ABCB1	ATP-binding cassette subfamily B member 1
AMPK	Adenosine monophosphate-activated protein kinase
APL	Acute promyelocytic leukemia
ASK1	Apoptosis signal-regulating kinase 1
ATF4	Activating transcription factor-4
ATF6	Activating transcription factor 6
ATO	Arsenic trioxide
BiP	Binding immunoglobulin protein
BTK inhibitors	Bruton’s tyrosine kinase inhibitors
CHOP	C/EBP homologous protein
COMET	Combination Organelle Mitochondrial Endoplasmic Reticulum Therapy
CuET	Bis (diethyldithiocarbamate)-copper
DFS	(−)-(2R, 3R)-1,4-O-diferuloylsecoisolariciresinol
DON	Diazo-5-oxo-L-norleucine
DPAGT1	UDP-N-acetylglucosamine–dolichol phosphate N-acetylglucosamine-1-phosphate transferase
DR4	Death receptor 4
DR5	Death receptor 5
EDEM1	ER degradation-enhancing α-mannosidase-like protein 1
EGCG	Epigallocatechin-3-gallate
eIF2α	Eukaryotic translation initiation factor 2α
EMT	Epithelial to mesenchymal transition
ER	Endoplasmic reticulum
ERAD	ER-associated degradation
FLCs	Free light chains
GFAT	Glutamine:fructose-6-phosphate amidotransferase
GRP78	Glucose-Regulated Protein 78
HDAC inhibitor	Histone deacetylase inhibitor
HDAC6	Histone deacetylase 6
HNK	Honokiol
Hsp70	Heat Shock Protein 70
HSP90	Heat Shock Protein 90
HSPA5	Heat Shock Protein Family A (Hsp70) Member 5 gene
IAP	Inhibitor of Apoptosis
IRE1α	Inositol-requiring enzyme 1 alpha
ISR	Integrated Stress Response
JNK	c-Jun N-terminal kinase
MCL	Mantle cell lymphoma
MDR	Multidrug-resistant
MFN2	Mitofusin 2
MiND	Mitochondrial Network Disrupting Nanoparticles
MM	Multiple myeloma
MTA	Multitargeted antifolate
Nrf1/NFE2L1	Nuclear factor erythroid 2-related factor 1
OXPHOS	Oxidative phosphorylation
PDI inhibitors	Protein disulfide isomerase inhibitors
PDIA6	Protein disulphide isomerase-associated 6
PERK	Protein kinase RNA-like endoplasmic reticulum kinase
P-gp	P-glycoprotein
PI	Proteasome inhibitor
PML	Promyelocytic Leukemia protein
PRISMA	Preferred Reporting Items for Systematic Reviews and Meta-Analyses
RIDD	Regulated IRE1α-dependent decay
ROS	Reactive oxygen species
S2P	Site-2-protease
SERCA	sarcoplasmic/endoplasmic reticulum Ca^2+^-ATPase
TKI	Tyrosine kinase inhibitor
TME	Tumor microenvironment
TMZ	Temozolomide
TNBC	Triple Negative Breast Cancer
TRAF2	TNF receptor-associated factor 2
TRIB3	Tribbles 3
TRPV1	Transient Receptor Potential Vanilloid type 1
UPR	Unfolded Protein Response
UPS	Ubiquitin Proteasome System
XBP1	X-box binding protein 1
XN	Xanthohumol
